# The role of inflammation in the development of tic symptoms in subjects with ADHD

**DOI:** 10.1016/j.bbih.2025.100981

**Published:** 2025-03-22

**Authors:** Nagahide Takahashi, Hidekazu Kato, Yoshihiro Nawa, Shiori Ogawa, Kenji J. Tsuchiya, Takashi Okada

**Affiliations:** a Department of Developmental Disorders, National Center of Neurology and Psychiatry, Japan; b Research Center for Child Mental Development, Hamamatsu University School of Medicine, Japan; c United Graduate School of Child Development, The Osaka University, Kanazawa University, Hamamatsu University School of Medicine, Chiba University and University of Fukui, Japan; d Department of Child and Adolescent Psychiatry, Nagoya University Hospital, Japan; e Research Center of Health, Physical Fitness and Sports, Nagoya University, Japan; f Department of Psychiatry, Nara Medical University, Japan

**Keywords:** Attention deficit hyperactivity disorder, Tourette's syndrome, Neuroinflammation, Genome wide association study

## Abstract

Tourette's syndrome is characterized by multiple motor and 1 or more vocal tics that persist for more than 1 year since first tic onset. It is well known that subjects with Tourette's syndrome show varieties of comorbidities, and ADHD is one of the most prevalent comorbid symptoms. In most cases, ADHD symptoms is known to precede the onset of tic symptoms, but how subjects with ADHD develop Tourette's syndrome later in life remains unclear.

Both Tourette's syndrome and ADHD is highly heritable, and genome wide association studies of ADHD and Tourette's syndrome showed that Tourette's syndrome and ADHD are genetically related. In order to identify the factor to cause tic symptoms in subjects with ADHD, we conducted two-sample mendelian randomization analysis, gene-set analysis and identified *neutrophil degranulation* is a pathways specific to Tourette's syndrome. Molecular analysis showed that Neutrophil-lymphocyte ratio may be relatively upregulated within the normal range in subjects with ADHD and Tourette's syndrome compared to subjects with ADHD only.

As the molecular analysis is still in its preliminary stages, the current results suggest that inflammation may be a contributing factor in the development of symptoms of Tourette's syndrome in subjects with ADHD. If these results can be replicated, neutrophil-lymphocyte ratio could serve as a potential a biomarker for the risk of Tourette's syndrome.

## Highlights

• ADHD is one of the most prevalent comorbid conditions of Tourette's syndrome. ADHD symptoms is known to precede the onset of tic symptoms, but how subjects with ADHD develop Tourette's syndrome later in life remains unclear.
• Gene-set analysis using genome wide studies of ADHD and Tourette's syndrome identified *neutrophil degranulation* is a pathway specific to Tourette's syndrome. Molecular analysis showed that the neutrophil-lymphocyte ratio was upregulated in subjects with ADHD and Tourette's syndrome compared to subjects with ADHD alone, with no effect of ADHD medication and antipsychotics.
• Highly activated inflammation added to the baseline inflammation in ADHD is suggested to be implicated in the development of symptoms of Tourette's syndrome in ADHD subjects.
• Neutrophil-lymphocyte ratio can be a biomarker for the risk of Tourette's syndrome and neuroinflammation may be a potential target for controlling symptoms in Tourette's syndrome.

## Graphical abstract

**Figure:**
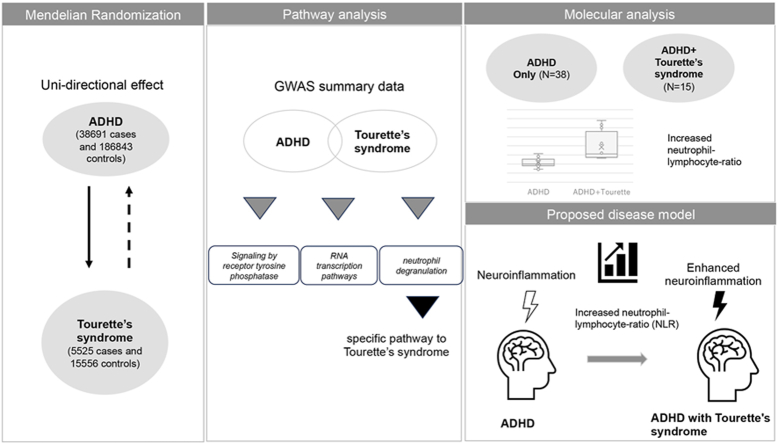

## 1 Introduction

Tourette's syndrome is characterized by continuous vocal and motor tick. It is well known that subjects with Tourette's syndrome show varieties of comorbidities, but ADHD is one of the most prevalent comorbid symptoms. In subjects with Tourette's syndrome and ADHD, it has been reported that ADHD symptoms precede the onset of tic symptoms (Mol Debes, 2013).

Since both Tourette's syndrome and ADHD is highly heritable, genome wide association studies (GWAS) of ADHD (Demontis et al., 2023) and Tourette's syndrome (Yu et al., 2019) have been conducted and several genomic loci have been identified as risk loci for each disorder. However, it has not been clarified how subjects with ADHD develop Tourette's syndrome later in life.

Therefore, we hypothesized that although Tourette's syndrome and ADHD are genetically related (Cross-Disorder Group of the Psychiatric Genomics Consortium, 2019), there may be a different genetic background between ADHD and ADHD with Tourette's syndrome which can be a trigger to cause tic symptoms in subjects with ADHD, which eventually develop continuous motor, and vocal tics diagnosed as ADHD and Tourette's syndrome. To test this hypothesis, we first conducted two-sample mendelian randomization analysis, gene-set analysis to identify pathways specific to Tourette's syndrome and molecular analysis based on the results of gene-set analysis (Supplementary Fig. 1 for schematic flow of the analysis).

## 2 Material and methods

Two-sample mendelian randomization analysis was conducted using TwoSampleMR (https://mrcieu.github.io/TwoSampleMR/) (Hemani et al., 2018) and MR-PRESSO (https://github.com/rondolab/MR-PRESSO.git) (Verbanck et al., 2018). MR-Egger and Inverse Variance Weighted (IVW) methods were used for MR analysis. MR-PRESSO was used for outlier test. We used the IVW method and MR-Egger regression to detect heterogeneity. The statistical power of the MR analysis was calculated using mRnd (https://shiny.cnsgenomics.com/mRnd/).

Gene-set analysis using summary data of GWAS of Tourette's syndrome and ADHD were performed to identify genetic pathways specific to Tourette's syndrome but not to ADHD. Summary data of GWAS of ADHD and Tourette's syndrome were obtained from PGC (https://pgc.unc.edu/for-researchers/download-results/). Gene mapping of SNPs obtained from the summary data of each GWAS was conducted using positional mapping, eQTL mapping and Chromatin interaction mapping using FUMA (https://fuma.ctglab.nl) (Watanabe et al., 2017). MsigDB (https://www.gsea-msigdb.org/gsea/index.jsp) was used to identify gene-sets associated with the diseases, focusing on all Canonical Pathways including BioCarta, KEGG and Reactome (MsigDB c2). Using the hypergeometric distribution, a p-value of gene overlap was calculated and corrected for multiple hypothesis testing according to Benjamini and Hochberg using MAGMA gene-set analysis (de Leeuw et al., 2015).

Molecular analysis was conducted to examine if there was a difference in the molecules related to genetic pathways specific to Tourette's syndrome but not ADHD using serum obtained from subjects with ADHD (N = 43) and ADHD with Tourett's (N =25) syndrome. We recruited a convenience sample from patients attending Nagoya University Hospital. Patients with a history of any physical illness or recent infection were excluded. Diagnosis of ADHD and Tourette's syndrome were made by board certified child psychiatrist according to DSM-5. The neutrophil-to-lymphocyte ratio (NLR) was calculated as a ratio between the neutrophil and lymphocyte counts measured in peripheral blood. Group comparison was conducted using un-paired t-tests using Stata version 16. All statistical analysis was two-tailed.

The study protocol was approved by the Nagoya University Hospital Ethics Committee (Ref No. 2024-0153). This study followed the STrengthening the Reporting of OBservational studies in Epidemiology (STROBE) reporting guidelines and Sex and Gender Equity in Research (SAGER) guidelines.

## 3 Results

MR analysis showed that the MR-Egger and Inverse Variance Weighted (IVW) methods both indicated a significant uni-directional association between ADHD diagnosis and Tourette's syndrome (ADHD - > Tourette's syndrome: MR-Egger, beta [SE] = 1.369[0.329], P = 4.40x10^−5^; IVW, beta[SE] = 0.971[0.001], P = 2.418x10^−31^; Tourette's syndrome - > ADHD: MR Egger beta [SE] = 0.071[0.007], P = 0.3625; IVW, beta[SE] = 0.004[0.008], P = 0.591) (Table 1). The heterogeneity statistics demonstrated no heterogeneity among these studies. Furthermore, the statistical power of the Tourette's syndrome GWAS was 0.98, suggesting that type II error is unlikely in the results of the MR analysis between Tourette's syndrome and ADHD.

**Table 1:** Results of two sample mendelian randomization analysis.

ADHD - > TS	SNP	B	se	P-value
MR -Eggar	244	1.369	0.329	4.400x10^−5^
IVW	244	0.9706	0.0005954	2.418x10^−31^

**TS- > ADHD**	**SNP**	**B**	**se**	**P-value**

MR -Eggar	13	0.07129	0.007504	0.3625
IVW	13	0.004754	0.008621	0.5914

Mendelian randomization analysis showed a significant uni-directional association between ADHD diagnosis and Tourette's syndrome. **Abbreviations:** ADHD, attention deficit hyperactivity disorder; TS, Tourette's syndrome; SNP, single nucleotide polymorphism, B, standardized beta; SE, standard error; MR-eggar, Mendelian Randomization; IVW, Inverse variance weighted.

Since only two Reactome pathways, “*RNA transcription pathways”* and “*neutrophil degranulation”* have been identified as related to Tourette's syndrome by gene-sets analysis using MsigDB (See Supplementary Fig. 2), we only focused on Reactome pathways in the gene-sets analysis of ADHD and two Reactome pathways “*Signaling by receptor tyrosine phosphatase”* and “*RNA transcription pathways”* were found to be associated with ADHD after FDR correction (See Supplementary Table 1 for the list of SNPs and corresponding genes and Supplementary Table 2 for the results of all pathway analyses). Therefore,*“RNA transcription pathways”* are common to both Tourette and ADHD, and “*neutrophil degranulation”* was specific to Tourette's syndrome.

**Fig. 1:**
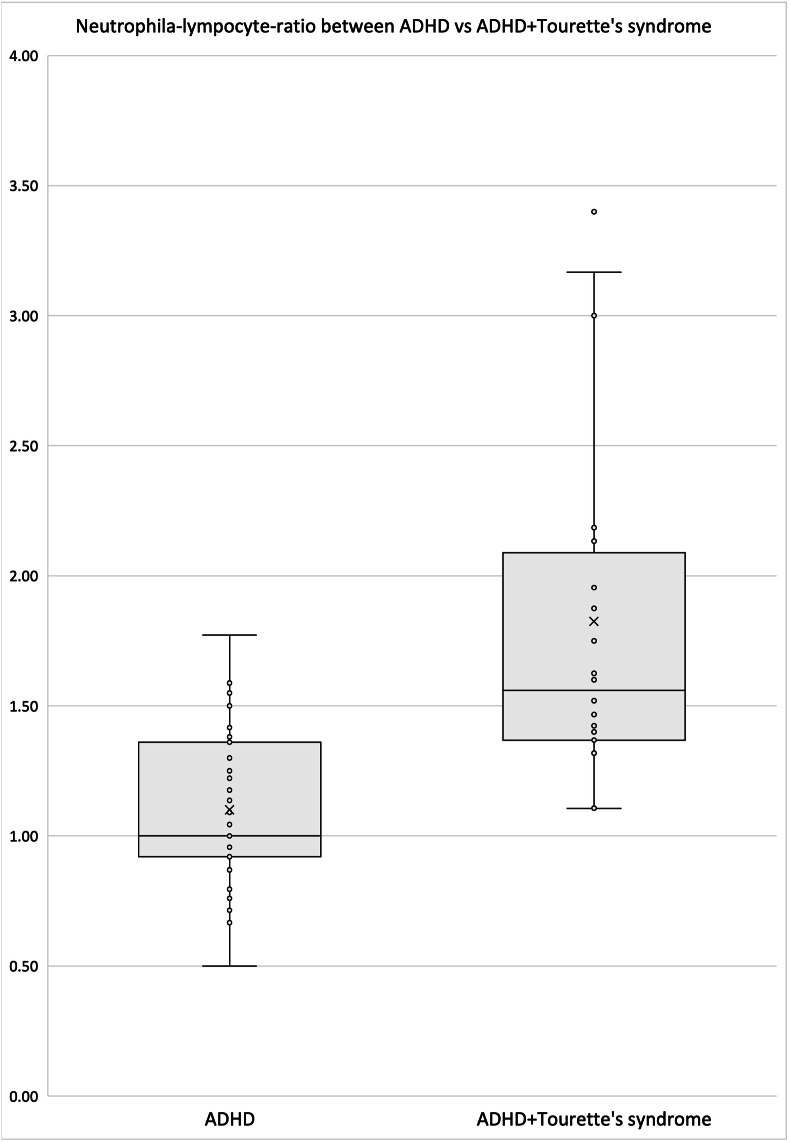
Neutrophil-lymphocyte-ratio between ADHD (N = 43) vs ADHD with Tourette's (N = 25). Box plots of Neutrophil-lymphocyte-ratio in subjects with ADHD and subjects with ADHD and Tourette's syndrome are shown. **Abbreviations:** ADHD, attention deficit hyperactivity disorder; TS, Tourette's syndrome.

Several lines of evidence suggested that neuroinflammation is implicated in the mechanism of neurodevelopmental disorders (Han et al., 2021). Therefore, we hypothesized that activation of neuroinflammation characterized by neutrophil pathways can trigger Tourette's syndrome in ADHD subjects. Neutrophil-lymphocyte ratio (NLR) has been proposed as an indicator of neuroinflammation (Bhikram and Sandor, 2022), thus we compared the NLR between ADHD only group (N = 43; 29 Males and 14 females; mean age [SD] = 13.684 [5.014]) and ADHD + Tourette's syndrome group (N = 25; 23 males and 2 females; mean age [SD] = 14.174 [6.249]). We found that NLR is significantly increased in the ADHD + Tourette's syndrome group compared to the ADHD only group (t = −5.166, df = 65, P < 0.001, Fig. 1). Multiple regression analysis showed that NLR is significantly increased after controlling age and sex.

Since there has been some reports which showed that medications for ADHD and antipsychotics may modulate neuroinflammation (Patel et al., 2022), we examined whether the use of ADHD medication affect NLR and whether there is an association between chlorpromazine equivalent dose of antipsychotics and NLR in subjects with ADHD + Tourette's syndrome. We found that there is no difference in NLR between anti-ADHD medicated and anti-ADHD unmedicated subjects with ADHD + Tourette's syndrome (t = −0.2269, df = 23, P = 0.824; Supplementary Fig. 3a). Furthermore, there was no association between the dose of antipsychotics and NLR in subjects with ADHD + Tourette's syndrome (beta [SE] = 0.0122[0.001], P = 0.201, Supplementary Fig. 3b).

## 4 Discussion

The present study showed that genetic components associated with ADHD can also be a risk for developing Tourette's syndrome and pathways related to inflammation induced by neutrophil activation may trigger tic symptoms in subjects with ADHD. The results can partially explain why ADHD symptoms precede motor and vocal tics in Tourette's syndrome. Although antipsychotics are frequently used for treating symptoms of Tourette's syndrome, around 30 % of subjects with Tourette's syndrome are treatment resistant and does not respond to antipsychotics. Therefore, neuroinflammation may be a promising target for intervention to improve tic symptoms in Tourette's syndrome.

The present study is consistent with the previous study which showed genetic correlation between ADHD and Tourette's syndrome (Patel et al., 2022). However, there has been no study reporting unique gene-sets for each of the disorders. Therefore, we believe that this is the first study to show that there is a unique pathway, “*neutrophil degranulation”*, specific to Tourette's syndrome. Neutrophils degranulation is considered to be a response to inflammation and neutrophil degranulation itself can also promote further recruitment and activation of neutrophils, which is observed as increased NLR (Bai et al., 2021).

Increased NLR has been repeatedly reported in subjects with ADHD and the actual figures of NLR were consistent with those observed in previous studies (Gedek et al., 2023). However, there has been no reports investigating NLR in Tourette's syndrome. To our knowledge, this is the first study showed increased NLR in subjects with ADHD and Tourette's syndrome compared to subjects with ADHD only. As neuroinflammation is implicated in both ADHD and Tourette's syndrome (Han et al., 2021), the symptoms of Tourette's syndrome in ADHD subjects can be attributed to a further, highly activated inflammation added to the baseline inflammation in ADHD. More recently, there have been reports suggesting direct evidence of an interaction between neutrophils and dopamine, which is implicated in the pathology of ADHD and Tourette's syndrome (Channer et al., 2023).

Although still controversial, it has been proposed that medications used for mental disorder have anti- or pro-inflammatory properties (Patel et al., 2022). For example, high dose of methylphenidate induce inflammation in animal models, whereas therapeutic dose of methylphenidate may suppress inflammation by activating kynurenic acid pathways. On the other hand, antipsychotics which often used for the treatment of symptoms in Tourette's syndrome are reported to reduce inflammatory cytokines. However, our data demonstrated that upregulation of NLR is independent of the use of anti-ADHD medication or antipsychotics, but dependent on the presence of diagnosis of Tourette's syndrome. Poor quality of sleep is also known to worsen symptoms of Tourette's syndrome (Ricketts et al., 2023), but melatonin has been reported to suppress neutrophil activation, which may be beneficial for controlling symptoms in subjects with Tourette's syndrome (Aslam et al., 2023).

There are couple of limitation in this study. First, due to the low prevalence of Tourette's syndrome, the number of subjects used in the NLR analysis was limited. Therefore, the results of this study need to be replicated with an independent sample. Second, since the number of subjects analyzed in the original GWAS study is smaller in Tourette's syndrome compared to that of ADHD, there might be other gene-sets, especially brain-specific pathways, which are specific to Tourette's syndrome, but not for ADHD. If so, molecular pathways other than inflammation can differentiate ADHD and ADHD with Tourette's syndrome. Third, although NLR has been reported as surrogate marker for neuroinflammation, there is a possibility that NLR does not directly reflect inflammation in the brain. Lastly, since we did not evaluate the severity in symptoms of ADHD nor Tourette's syndrome in subjects used for molecular analysis, the usefulness of NLR as a symptomatic biomarker remains unknown. In addition, due to the lack of control subjects in this study, it is difficult to assess whether there is any evidence of inflammation in peripheral blood. Future studies using large samples with detailed information regarding disease severity and longitudinal data are needed to clarify the causal role of inflammation on these conditions.

## 5 Conclusion

As the molecular analysis is still in its preliminary stages, the current results suggest that inflammation may be a contributing factor in the development of symptoms of Tourette's syndrome in subjects with ADHD. If these results can be replicated, the neutrophil lymphocyte ratio could serve as a potential biomarker for the risk of Tourette's syndrome.Further studies using an independent sample set are warranted.

## CRediT authorship contribution statement

**Nagahide Takahashi:** Writing – review & editing, Writing – original draft, Project administration, Methodology, Formal analysis, Data curation, Conceptualization. **Hidekazu Kato:** Writing – review & editing, Project administration, Data curation, Conceptualization. **Yoshihiro Nawa:** Writing – review & editing, Project administration, Data curation. **Shiori Ogawa:** Writing – review & editing, Writing – original draft, Conceptualization. **Kenji J. Tsuchiya:** Funding acquisition, Writing – review & editing, Writing – original draft, Formal analysis, Data curation, Conceptualization. **Takashi Okada:** Writing – review & editing, Supervision, Funding acquisition, Conceptualization.

## Declaration of generative AI in scientific writing

None.

## Funding/support

This work was supported by grants from the Ministry of Education, Culture, Sports, Science, and Technology in Japan (grant number 19H03582 to KJT).

## Declaration of competing interest

Dr. Takahashi reported receiving research grants from the Japan Society for the Promotion of Science (JSPS) (21K07479, 21H02848, and 19H03582), the Japan Agency for Medical Research and Development (AMED) (20dk0307094h0001), as well as personal fees from Janssen, Takeda, Otsuka, Shionogi, and Nobel Pharma for lectures. He also owns a stake in Johnson & Johnson. Dr. Tsuchiya reported receiving research grants from JSPS (19H03582, 19K10997, 20K02628, 20K07941, 21K19639 and 21KK0145) and AMED (JP21gk0110039h0003), speakers’ honoraria from Sumitomo Dainippon, and has served as an advisor to the City of Hamamatsu. Dr. Okada reported receiving research grants from the Japan Society for the Promotion of Science (JSPS) (20K07917) as well as personal fees from Janssen, Takeda, Yoshitomi, Shionogi, and Nobel Pharma for lectures.

## Data Availability

The authors do not have permission to share data.
