# Supplementary material for: The role of inflammation in the development of tic symptoms in subjects with ADHD
Source: Brain Behav Immun Health. 2025 Mar 22;45:100981. doi: 10.1016/j.bbih.2025.100981 (PMC11994945; doi:10.1016/j.bbih.2025.100981)
Supplement: Multimedia component 1 [file mmc1.docx]

**Supplementary Figure 1. Schematic flow of the analysis**

**
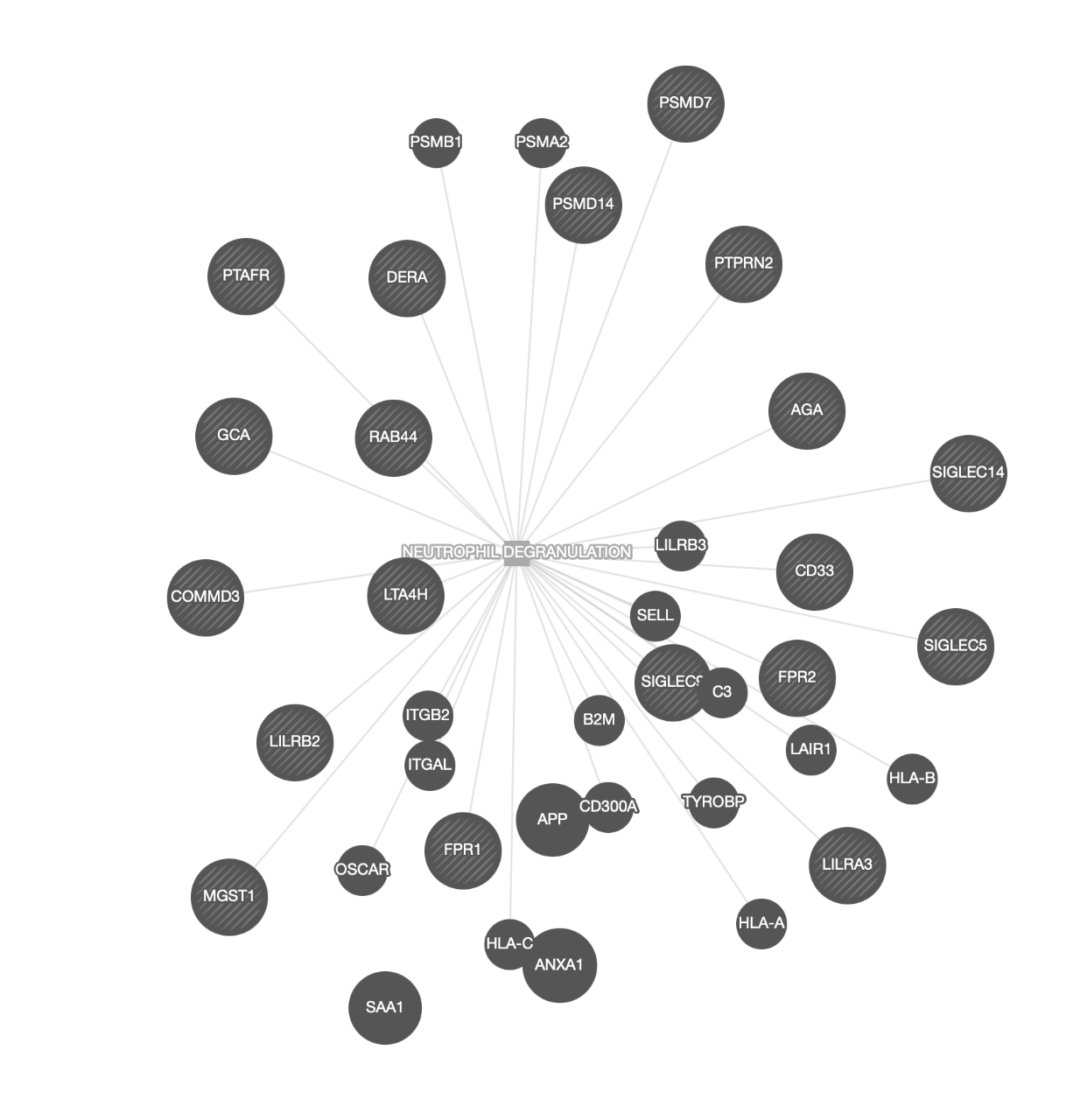
Supplementary Figure 2. Gene enrichment analysis of genes associated with Tourette syndrome**.

GENEMANIA(<https://genemania.org>) was used to visualize gene network.

**Supplementary Figure 3. Effect of ADHD medication and antipsychotics on Neutrophil-lymphocyte-ratio.**

1. **Effect of ADHD medication and antipsychotics on Neutrophil-lymphocyte-ratio.**


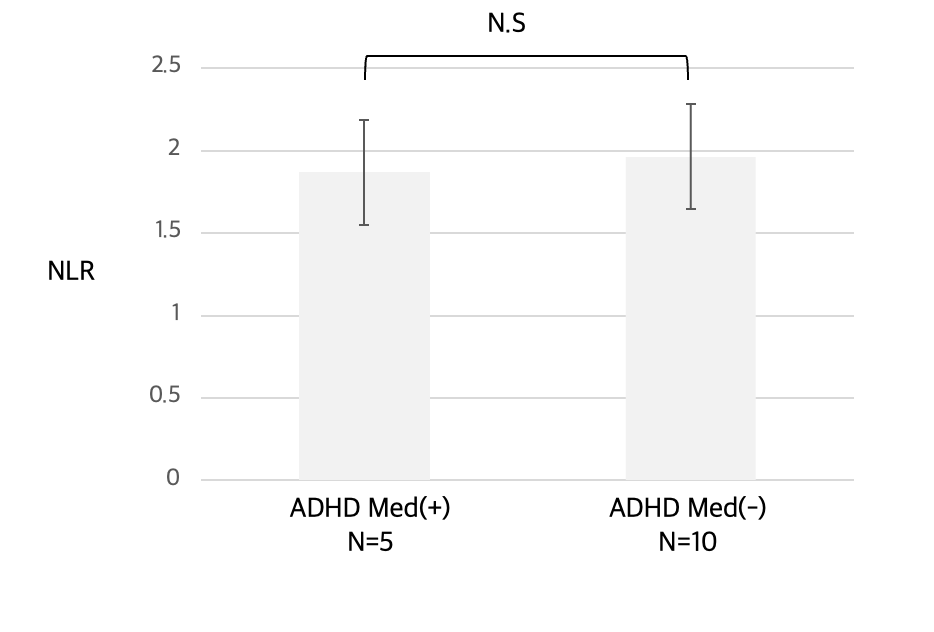


1. **Effect of antipsychotics on Neutrophil-lymphocyte-ratio.**
